# Supplementary figures and images for: Recovering genotypes and phenotypes using allele-specific genes
Source: Genome Biol. 2021 Sep 7;22:263. doi: 10.1186/s13059-021-02477-x (PMC8425091; doi:10.1186/s13059-021-02477-x)

Fig. S1

Case P-P :

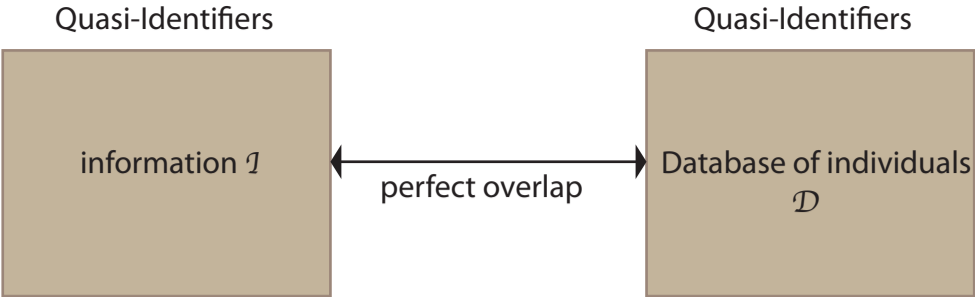

Case P-N

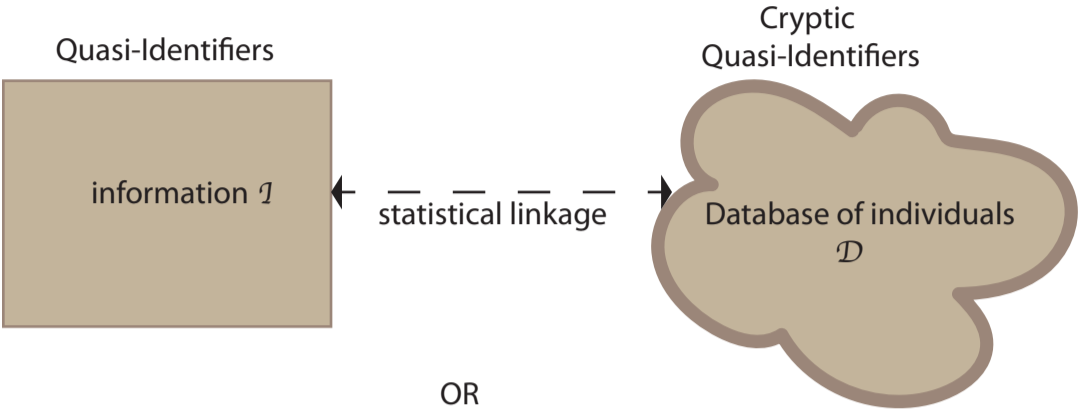

or

N-P :

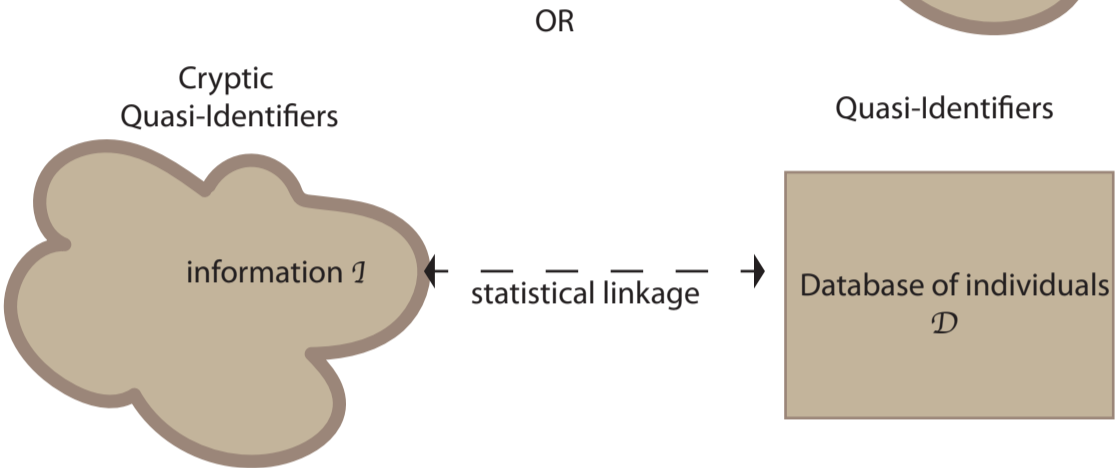

Case N-N:

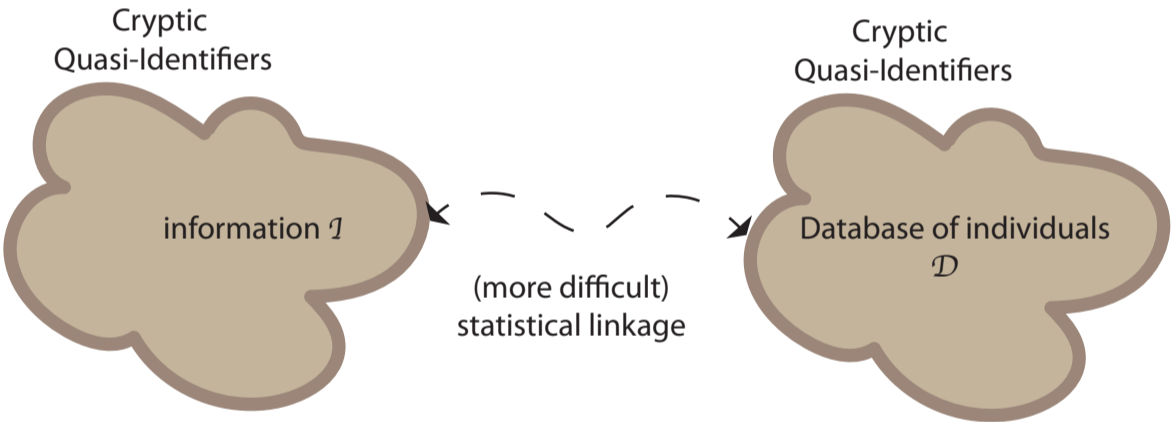

Case P-N\*

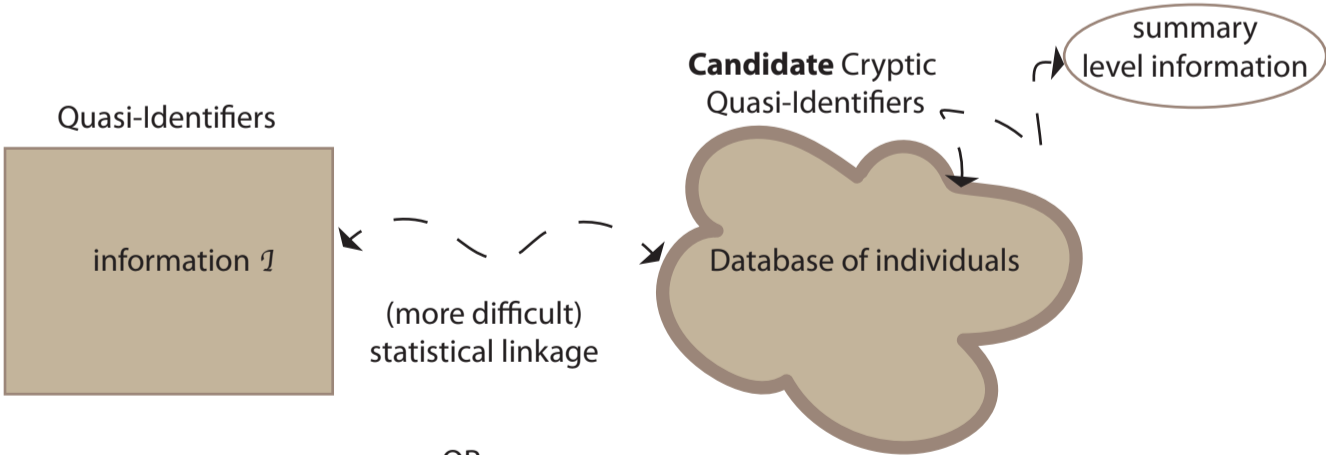

or

N\*-P :

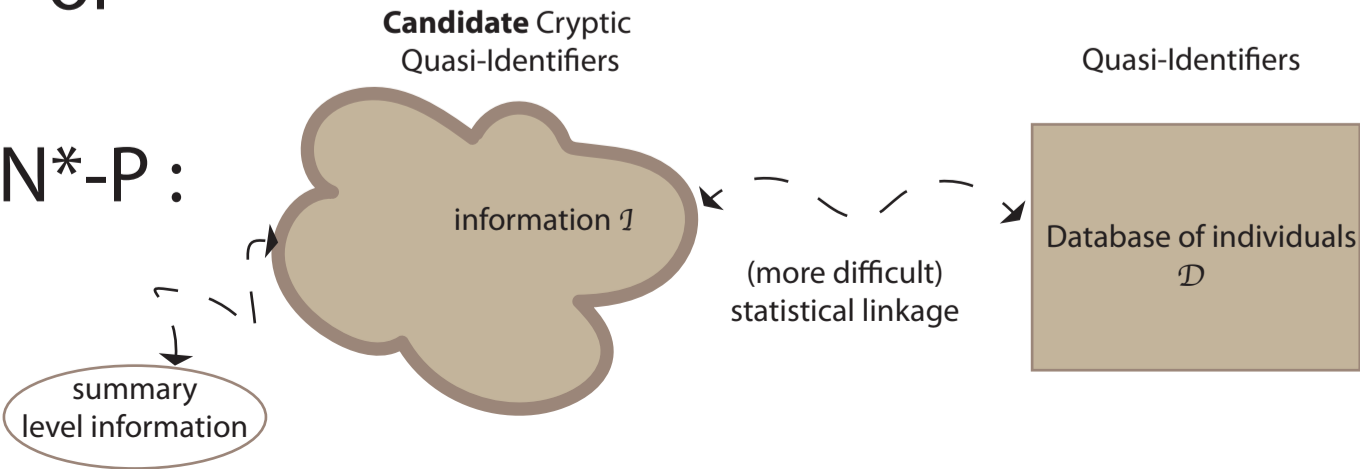

Supplement: Supplementary file 1 — Additional file 1: Figure S1. Different cases of linkage attacks. [file 13059_2021_2477_MOESM1_ESM.pdf]

Fig. S2

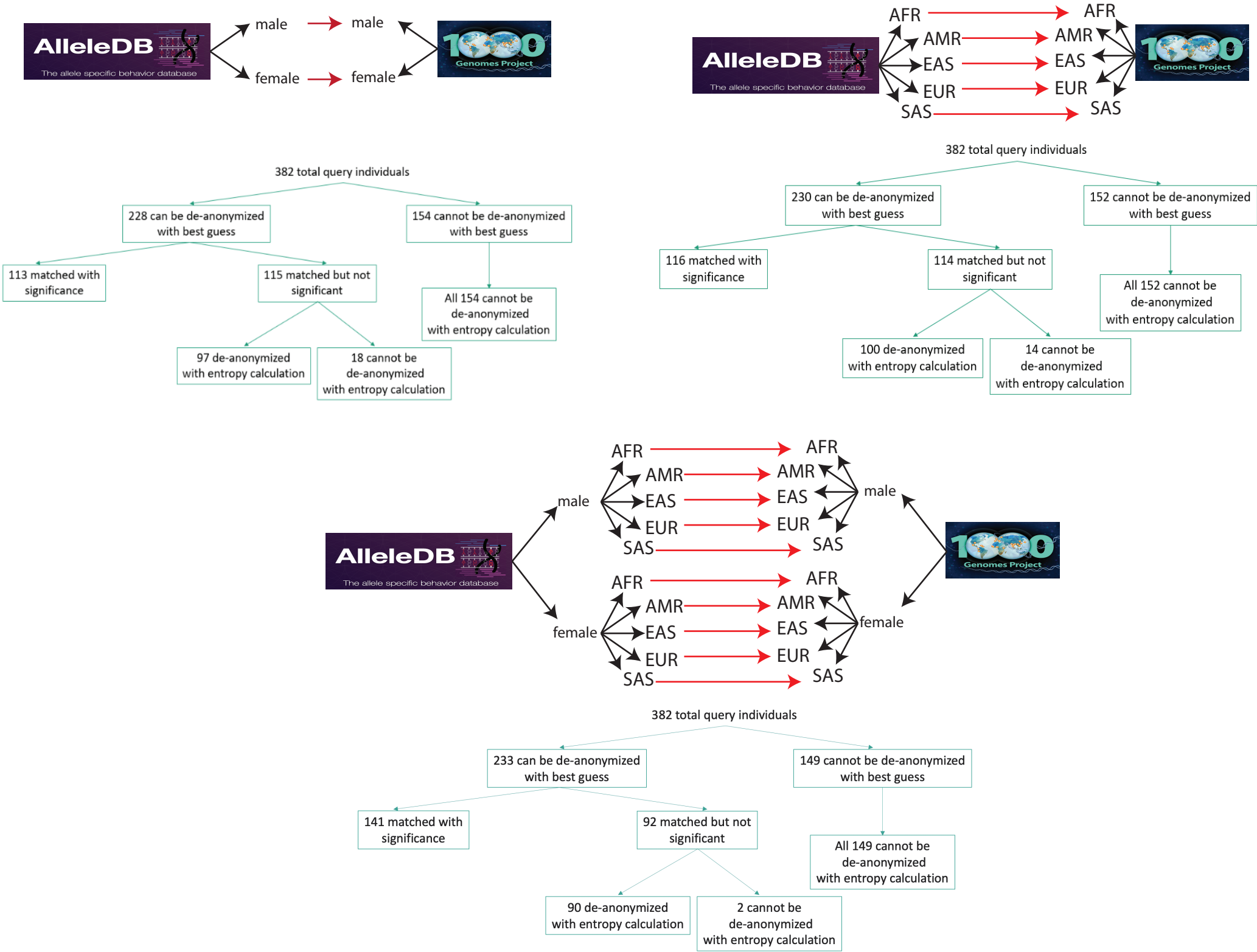

Supplement: Supplementary file 3 — Additional file 3: Figure S2. Number of individuals that are correctly identified using different methodologies and different auxiliary information. [file 13059_2021_2477_MOESM3_ESM.pdf]

# Fig. S3

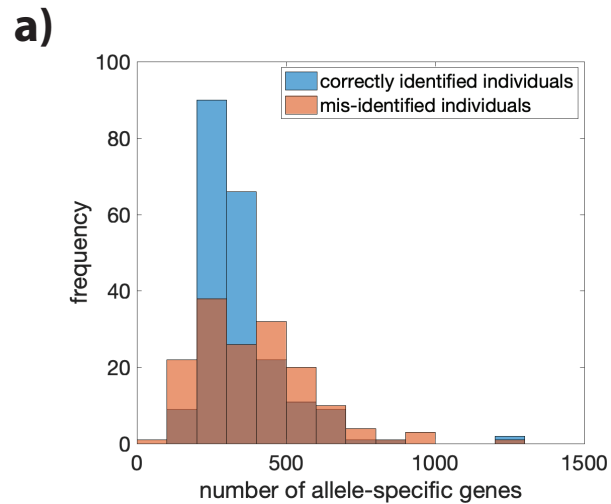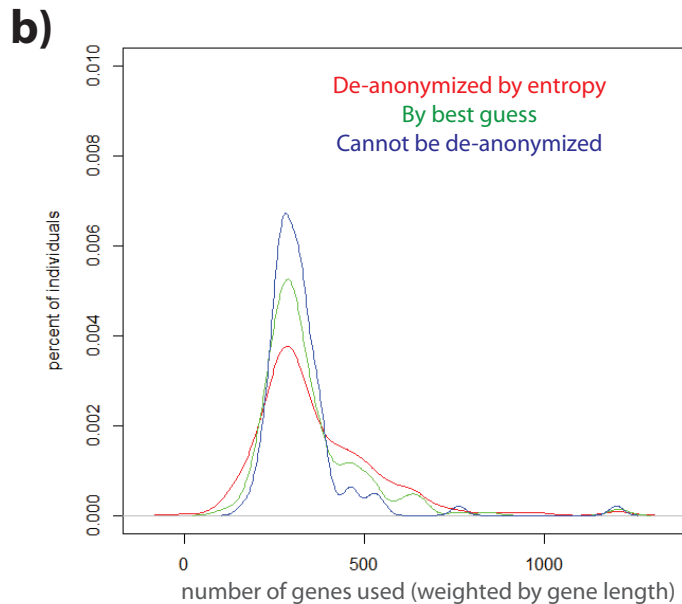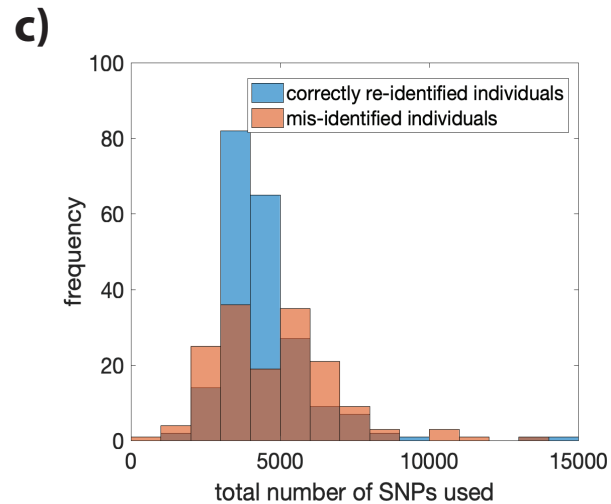

Supplement: Supplementary file 4 — Additional file 4: Figure S3. a) Distribution of the number of ASE genes used for correctly identified individuals (in blue) and for misidentified individuals (in orange). b) Same as (a), but the genes are weighted by their length. c) Distribution of number of candidate SNPs inferred for correctly identified individuals (in blue) and for mis-identified individuals (in orange). [file 13059_2021_2477_MOESM4_ESM.pdf]

# Fig. S4

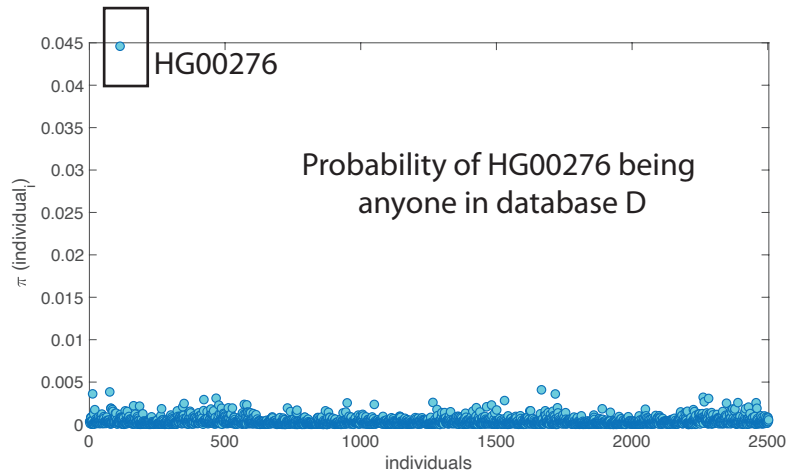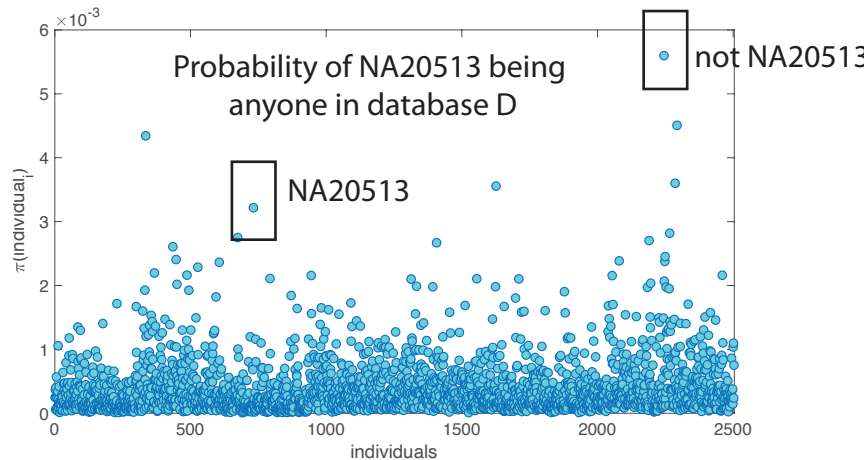

Supplement: Supplementary file 5 — Additional file 5: Figure S4. Examples of entropy-based matching for a correctly identified individual (HG00276) and mis-identified individual (NA20513). [file 13059_2021_2477_MOESM5_ESM.pdf]
