# Supplementary material for: Recovering genotypes and phenotypes using allele-specific genes
Source: Genome Biol. 2021 Sep 7;22:263. doi: 10.1186/s13059-021-02477-x (PMC8425091; doi:10.1186/s13059-021-02477-x)
Supplement: Supplementary file 2 — Additional file 2. Supplementary Information for Recovering genotypes and phenotypes using allele-specific genes. [file 13059_2021_2477_MOESM2_ESM.pdf]

Supplementary Information

Calculating precision, recall, and false positive rate

The attacker does not assume the victim is in the database. The assumption is that if the score of the top ranked match is different than the distribution of the scores for other individuals, then the top ranked match is the victim. If the score is not different from, say, the second ranked individual, the victim is not in the database. However, there might be significant matches that are incorrect.

Below how we calculated the accuracy metrics

1- We can correctly match the individual, who is in the database. This counts as a true positive, which is equal to 211 individuals.

2- The individual is in the database but we don't have a match (i.e score that is different from the score of the second best match). This counts as false negative, which is equal to 171 individuals.

3- We removed the individuals from the database one by one and performed the linking. If there is no match then that counts towards true negative, which is equal to 307 individuals.

4- We removed the individuals from the database one by one and performed the linking. If there is a match then that counts towards false positive, which is equal to 75 individuals.

This gives us the following values for recall, FPR, and precision:

Recall = tp/(tp+fn) = 0.55
False positive rate = fp/(fp+tn) = 0.20
Precision = tp/(tp+fp) = 0.74

Below are the values when we removed the HLA genes:

TP=251, FN=131, FP= 36, and TN = 246
Recall = 0.66
FPR = 0.09
Precision = 0.87

Below are the values when we removed the top 20 overlapping genes:

TP=260, FN=122, FP= 28, and TN = 354
Recall = 0.68
FPR = 0.07

Precision = 0.91

Genotyping frequency threshold for SNP selection

If a heterozygous SNP has a genotyping frequency smaller than 0.1, then it means that the alternative allele is present in a small number of individuals, which makes it very informative if we calculate the self information (>$-\log_2(0.1)$). Although this is very useful in linking individuals to a database when we know "for sure" that the alternative allele is present in the victim's genome. However, in our scenario, the attacker makes predictions of the presence of the rare allele purely based on the fact that if the gene has ASE or not. The attacker will more likely be wrong in guessing it than being right. If the attacker falsely predicts the presence of an alternative allele that is very rare in the population, the resulting linking score will be dominated by the contribution of this rare SNP and, in turn, will lead to linking the ASE to the wrong individual. Therefore these SNPs are removed from the list.

If a heterozygous SNP has a genotyping frequency larger than 0.5, then it means it is common in the population. Therefore, it's contribution to the linking score is 1 ($-\log_2(0.5)$) or smaller. Adding this criteria to the predicted SNPs helps attackers weed out the non-informative SNPs from the list for a quicker calculation. Therefore these SNPs are removed from the list.

We have calculated how the thresholding affects the linking ability. We found that when there is no thresholding, the accuracy drops from 55% to 41%. When we only remove the rare ones (<0.1 genotyping frequency) and leave the ones with >0.5 genotyping accuracy, our accuracy is 55%, suggesting the removal of common SNPs do not affect the linking ability but makes the calculation faster by reducing the number of SNPs in the list. Below are different threshold and the number of correctly individuals:

No threshold = 157/382 = 0.41
Remove only rare (<0.05) = 90/382 = 0.24
Remove only rare (<0.1) = 211/382 = 0.55
Remove only rare (<0.2) = 173/382 = 0.45
Remove only rare (<0.1) and common (>0.5) = 211/382 = 0.55

Self-information of ASE genes

We calculated how many individuals (n_g) a gene is observed as ASE among all 382 individuals and calculated the amount of self-information a gene contains as:

$$I(g) = -\log_2 (n_g/382)$$

As can be seen from Figure 2x and as expected, the genes that are ASE for a fewer number of individuals are more informative, which means we can infer more unique SNPs by using them. On the other hand, they are more informative for fewer individuals..
