# Supplementary material for: Recovering genotypes and phenotypes using allele-specific genes
Source: Genome Biol. 2021 Sep 7;22:263. doi: 10.1186/s13059-021-02477-x (PMC8425091; doi:10.1186/s13059-021-02477-x)
Supplement: Supplementary file 6 — Additional file 6. Review history. [file 13059_2021_2477_MOESM6_ESM.docx]

Review History

**First round of review**

**Reviewer 1**

**Were you able to assess all statistics in the manuscript, including the appropriateness of statistical tests used?**

Yes, and I have assessed the statistics in my report.

**Comments to author:**

The paper presented linking attacks to identify human individuals from shared allele specific expressed gene lists. It shows the presence of privacy risks of sharing summary results from the analyses of allele specific gene expression, even when only the gene names instead of specific variants are shared, and thus showed potential implications of sharing allele specific gene expression analyses results. Overall, the paper presented a novel approach, and the methodology is solid. I have a few comments of the paper that the authors may consider in their revision.

1. The assumption of the linking attack is not completely clear. The linking attack report the matched genome(s) in a database (based on the linking score) with the input set of allele specifically expressed genes (or the presence of the subset of SNPs indicated by this list). Does it mean that the attacker assumes the victim is present in the database? How can the attacker estimate the confidence of the attack, i.e., the top hit is indeed the victim? It will be useful to estimate the false positive rate of the attack.

2. It is unclear how it is handled in the linking attack when a gene contains multiple rare alleles. Are only one of them to be considered to be present in the victim?

3. p7, it seems the SNPs with MAFs between 0.1 and 0.5 were removed; Why is this necessary? Does it mean that if a gene does not contain rare minor alleles, then it can be safely shared for its allele specific expression? It seems the method can be emhanced by considering the haplotypes of all minor alleles (after reconstructing haplotypes for each gene), which will incorporate not only the rare minor alleles).

4. How will the method handle multi-allelic (instead of biallelic) genes such as HLA. The paper suggests HLA was always reported as a allele specific expression gene and removing HLA from the consideration enhanced the attack. Is it because that each individual contains some minor alleles in the HLA genes? In that case, perhaps multi-allelic genes should be excluded from the analyses?

**Reviewer 2**

**Were you able to assess all statistics in the manuscript, including the appropriateness of statistical tests used?**

Yes, and I have assessed the statistics in my report.

**Comments to author:**

In this work, authors present a novel information leakage with the release of a ASE genes of an individual. They present two attacks both of which rely on the fact that it is possible to link the ASE genes to genomes. of people and identify them or their sensitive phenotype.

I have. the following concerns on the paper:

1. Authors state that "ASE is a characteristic of expressing only one copy of a gene (maternal or paternal allele) .." but it rather refers to the imbalance of expressed copy.

2. It is not clear how "the adversary compiles a database of genomes from known individuals (e.g., those who participated in a research study), by using genetic genealogy databases such as GEDmatch." Please clarify.

3. Please indicate that both studies used in the experiment are concerned with 1k genomes. The following sentence sounds like they might or no overlap: "We used lists of allele-specific gene names from 382 individuals 6 and attempted to link them to a database of 2,504 individuals 15."

4. I think the current match rates are underwhelming (50-60%). Following my previous point, they are underwhelming especially when one considers the fact that the samples in the ASE set are all in the genome set (1k genomes). That is, the people with ASE will not always a have a publicly available genome data. I wonder how the performance would look like if the authors removed varying portions of the 382 people from the 2504 genomes and calculate precision and recall. I also think top-20 match is not practically useful at all.

5. It is not clear how authors calculate the significance levels. They refer to a previously defined gap measure but no references are given. Please also discuss here with notation.

6. I do not understand why adding both ancestry and gender results in a lower performance increase. Please discuss the reason.

7. The authors do not have a fully phased dataset, so the heterozygous SNP frequency calculation and filtering is an ad-hoc procedure. How sensitive is this attack to different filter thresholds?

8. What is the term "e" in the entropy? Also what is the purpose of using this method? Can't the authors already have a distribution from the linking scores?

**Authors Response**

**Point-by-point responses to the reviewers’ comments:**

Reviewer 1:

The paper presented linking attacks to identify human individuals from shared allele specific expressed gene lists. It shows the presence of privacy risks of sharing summary results from the analyses of allele specific gene expression, even when only the gene names instead of specific variants are shared, and thus showed potential implications of sharing allele specific gene expression analyses results. Overall, the paper presented a novel approach, and the methodology is solid. I have a few comments of the paper that the authors may consider in their revision.

*Response*

*We thank the reviewer for these encouraging comments. We hope that our responses below and subsequent revision are satisfactory for this reviewer.*

The assumption of the linking attack is not completely clear. The linking attack report the matched genome(s) in a database (based on the linking score) with the input set of allele specifically expressed genes (or the presence of the subset of SNPs indicated by this list). Does it mean that the attacker assumes the victim is present in the database? How can the attacker estimate the confidence of the attack, i.e., the top hit is indeed the victim? It will be useful to estimate the false positive rate of the attack.

*Response*

*We thank the reviewer for this comment.*

*The attacker does not assume the victim is in the database. The assumption is that if the score of the top ranked match is different than the distribution of the scores for other individuals, then the top ranked match is the victim. If the score is not different from, say, the second ranked individual, the victim is not in the database. However, there might be significant matches that are incorrect. Calculating the false positive rate is indeed a good idea.*

*Below how we calculated the confusion matrix*

*1- We can correctly match the individual, who is in the database. This counts as a true positive, which is equal to 211 individuals.*

*2- The individual is in the database but we don’t have a match (i.e score that is different from the score of the second best match). This counts as false negative, which is equal to 171 individuals.*

*3- We removed the individuals from the database one by one and performed the linking. If there is no match then that counts towards true negative, which is equal to 307 individuals*

*4- We removed the individuals from the database one by one and performed the linking. If there is a match then that counts towards false positive, which is equal to 75 individuals.*

*This gives us the following values for recall, FPR, and precision:*

*Recall = tp/(tp+fn) = 0.55*

*False positive rate = fp/(fp+tn) = 0.20*

*Precision = tp/(tp+fp) = 0.74*

*We have now added these calculations and associated text to the manuscript and supplementary information.*

*Excerpt From*

*Revised Manuscript*

*In main text*

*We also calculated the precision as 74% and false-positive rate as 20% (see Supplementary Info).*

*In supplement:*

*Calculating precision, recall, and false positive rate*

*The attacker does not assume the victim is in the database. The assumption is that if the score of the top ranked match is different than the distribution of the scores for other individuals, then the top ranked match is the victim. If the score is not different from, say, the second ranked individual, the victim is not in the database. However, there might be significant matches that are incorrect.*

*Below how we calculated the metrics*

*1- We can correctly match the individual, who is in the database. This counts as a true positive, which is equal to 211 individuals.*

*2- The individual is in the database but we don’t have a match (i.e score that is different from the score of the second best match). This counts as false negative, which is equal to 171 individuals.*

*3- We removed the individuals from the database one by one and performed the linking. If there is no match then that counts towards true negative, which is equal to 307 individuals*

*4- We removed the individuals from the database one by one and performed the linking. If there is a match then that counts towards false positive, which is equal to 75 individuals.*

*This gives us the following values for recall, FPR, and precision:*

*Recall = tp/(tp+fn) = 0.55*

*False positive rate = fp/(fp+tn) = 0.20*

*Precision = tp/(tp+fp) = 0.74*

It is unclear how it is handled in the linking attack when a gene contains multiple rare alleles. Are only one of them to be considered to be present in the victim?

*Response*

*We consider all the SNPs overlapping with any exon of the gene as long as there are enough individuals (more than 10% and less than 50% of the population) with a heterozygous genotype for the SNP. If a SNP has more than one alternative allele, all rare alleles are considered to be a candidate for that individual.*

*We have now added a sentence to the main text to clarify this issue.*

*Excerpt From*

*Revised Manuscript*

*Note that all the SNPs that overlap with the exons are used as long as their genotyping frequency matches the criteria. If a SNP has more than one alternative allele, all alternative alleles are considered to be a candidate SNP.*

p7, it seems the SNPs with MAFs between 0.1 and 0.5 were removed; Why is this necessary? Does it mean that if a gene does not contain rare minor alleles, then it can be safely shared for its allele specific expression? It seems the method can be emhanced by considering the haplotypes of all minor alleles (after reconstructing haplotypes for each gene), which will incorporate not only the rare minor alleles).

*Response*

*We thank the reviewer for this question. Below we detailed the logic behind this thresholding. We have also done testing by changing this threshold to show how this threshold affects the linking ability.*

*If a heterozygous SNP has a genotyping frequency smaller than 0.1, then it means that the alternative allele is present in a small number of individuals, which makes it very informative if we calculate the self information (>-log2(0.1)). Although this is very useful in linking individuals to a database when we know “for sure” that the alternative allele is present in the victim's genome. However, in our scenario, the attacker makes predictions of the presence of the rare allele purely based on the fact that if the gene has ASE or not. The attacker will more likely be wrong in guessing it than being right. If the attacker falsely predicts the presence of an alternative allele that is very rare in the population, the resulting linking score will be dominated by the contribution of this rare SNP and, in turn, will lead to linking the ASE to the wrong individual. Therefore these SNPs are removed from the list.*

*If a heterozygous SNP has a genotyping frequency larger than 0.5, then it means it is common in the population. Therefore, it’s contribution to the linking score is 1 (-log2(0.5)) or smaller. Adding this criteria to the predicted SNPs helps attackers weed out the non-informative SNPs from the list for a quicker calculation. Therefore these SNPs are removed from the list.*

*We have calculated how the thresholding affects the linking ability. We found that when there is no thresholding, the accuracy drops from 55% to 41%. When we only remove the rare ones (<0.1 genotyping frequency) and leave the ones with >0.5 genotyping accuracy, our accuracy is 55%, suggesting the removal of common SNPs do not affect the linking ability but makes the calculation faster by reducing the number of SNPs in the list. We have also tried other genotyping frequency thresholds to see the effect.*

*We have now explained this in the manuscript and added a table on how this criteria affects the overall linking ability.*

*Excerpt From*

*Revised Manuscript*

*In main text, online methods:*

*We then removed the SNPs that had 0.1< f(genotypeSNPi=1)< 0.5 from the overlap list (see Supplementary Information for the rationale).*

*In supplementary information:*

*Genotyping frequency threshold for SNP selection*

*If a heterozygous SNP has a genotyping frequency smaller than 0.1, then it means that the alternative allele is present in a small number of individuals, which makes it very informative if we calculate the self information (>-log2(0.1)). Although this is very useful in linking individuals to a database when we know “for sure” that the alternative allele is present in the victim's genome. However, in our scenario, the attacker makes predictions of the presence of the rare allele purely based on the fact that if the gene has ASE or not. The attacker will more likely be wrong in guessing it than being right. If the attacker falsely predicts the presence of an alternative allele that is very rare in the population, the resulting linking score will be dominated by the contribution of this rare SNP and, in turn, will lead to linking the ASE to the wrong individual. Therefore these SNPs are removed from the list.*

*If a heterozygous SNP has a genotyping frequency larger than 0.5, then it means it is common in the population. Therefore, it’s contribution to the linking score is 1 (-log2(0.5)) or smaller. Adding this criteria to the predicted SNPs helps attackers weed out the non-informative SNPs from the list for a quicker calculation. Therefore these SNPs are removed from the list.*

*We have calculated how the thresholding affects the linking ability. We found that when there is no thresholding, the accuracy drops from 55% to 41%. When we only remove the rare ones (<0.1 genotyping frequency) and leave the ones with >0.5 genotyping accuracy, our accuracy is 55%, suggesting the removal of common SNPs do not affect the linking ability but makes the calculation faster by reducing the number of SNPs in the list. Below are different threshold and the number of correctly individuals:*

*No threshold = 157/382 = 0.41*

*Remove only rare (<0.05) = 90/382 = 0.24*

*Remove only rare (<0.1) = 211/382 = 0.55*

*Remove only rare (<0.2) = 173/382 = 0.45*

*Remove only rare (<0.1) and common (>0.5) = 211/382 = 0.55*

How will the method handle multi-allelic (instead of biallelic) genes such as HLA. The paper suggests HLA was always reported as a allele specific expression gene and removing HLA from the consideration enhanced the attack. Is it because that each individual contains some minor alleles in the HLA genes? In that case, perhaps multi-allelic genes should be excluded from the analyses?

*Response*

*We thank the reviewer for this comment. As mentioned in the response to Ref 1.3, we have a SNP in the candidate SNP list for every alternative allele in the case of multi-allelic genes.*

*Although multiallelic sites contribute to why removing HLA from the consideration enhances the attack; it is mostly due to how we predict the candidate SNP list. If a gene is ASE for two individuals, then the candidate SNP list for both individuals are exactly the same - so it does not give any distinguishing power to the attack. Therefore, certain genes such as the multi-allelic or imprinted genes are ASE for the majority of individuals, hence they make the linking attack more difficult. We ranked the genes by the number of individuals they are observed to be ASE. We then removed the top 20 of them and performed the linking attacks for the 382 individuals again. We found that the percentage of correctly linked individuals increased to 68%.*

*We have now added sentences to the main text to make these observations more clear.*

*Excerpt From*

*Revised Manuscript*

*This shows that removal of multi-allelic and imprinted genes that are ASE for many individuals from the consideration will enhance the linking attack ability. The genes that are ASE for a smaller number of individuals are the most informative, but, as a trade-off, they distinguish fewer numbers of individuals (see SI, Figure 2d).*

Reviewer 2:

In this work, authors present a novel information leakage with the release of a ASE genes of an individual. They present two attacks both of which rely on the fact that it is possible to link the ASE genes to genomes. of people and identify them or their sensitive phenotype

*Response*

*We thank the reviewer for the suggested reviews. We hope that our responses below and subsequent revision are satisfactory for this reviewer.*

Authors state that "ASE is a characteristic of expressing only one copy of a gene (maternal or paternal allele) .." but it rather refers to the imbalance of expressed copy.

*Response*

*We thank the reviewer for this comment and we agree with the reviewer on the definition of ASE. We have now changed the related sentence.*

*Excerpt From*

*Revised Manuscript*

*ASE is a characteristic of having an imbalance in the quantity of expressed copy of a gene (maternal or paternal allele) and may lead to phenotypic variation.*

It is not clear how "the adversary compiles a database of genomes from known individuals (e.g., those who participated in a research study), by using genetic genealogy databases such as GEDmatch." Please clarify.

*Response*

*We thank the reviewer for this question.*

*The genomes of known individuals can be trivial to obtain using the publicly available genetic genealogy databases such as GEDmatch. The adversary basically searches for individuals in the GEDmatch and it returns their genomes. The adversary can then build their own database from these genomes by making a look-up table, in which the columns are the genotypes of all the SNPs found in these genomes.*

*We have now changed this sentence to better explain.*

*Excerpt From*

*Revised Manuscript*

*The adversary compiles a comprehensive set of SNP genotypes from known individuals (e.g., those who participated in a research study), by using genetic genealogy databases such as GEDmatch. This database is a look-up table in which the columns correspond to the genotypes of the SNPs of all the individuals (Figure 1b).*

Please indicate that both studies used in the experiment are concerned with 1k genomes. The following sentence sounds like they might or no overlap: "We used lists of allele-specific gene names from 382 individuals [6] and attempted to link them to a database of 2,504 individuals [15]."

*Response*

*The ASE gene lists are from 382 individuals that are present in 2504 individuals of the 1k genome.*

*We have now made this clear in the manuscript.*

*Excerpt From*

*Revised Manuscript*

*We used lists of allele-specific gene names from 382 individuals 6 and attempted to link them to a database of 2,504 individuals that includes these 382 individuals 15*

I think the current match rates are underwhelming (50-60%). Following my previous point, they are underwhelming especially when one considers the fact that the samples in the ASE set are all in the genome set (1k genomes). That is, the people with ASE will not always a have a publicly available genome data. I wonder how the performance would look like if the authors removed varying portions of the 382 people from the 2504 genomes and calculate precision and recall. I also think top-20 match is not practically useful at all.

*Response*

*We thank the reviewer for this suggestion. While we agree that we need to calculate precision and recall, we strongly disagree that 50-60% accuracy is underwhelming in this setting.*

*The random chance of finding the correct individual among 2504 individuals is 0.0004 (1/2504). Therefore, our baseline is 0.004% and we are able to link >50% of individuals.*

*For precision and recall calculations, below how we calculated the confusion matrix:*

*1- We can correctly match the individual, who is in the database. This counts as a true positive, which is equal to 211 individuals.*

*2- The individual is in the database but we don’t have a match (i.e score that is different from the score of the second best match). This counts as false negative, which is equal to 171 individuals.*

*3- We removed the individuals from the database one by one and performed the linking. If there is no match then that counts towards true negative, which is equal to 307 individuals.*

*4- We removed the individuals from the database one by one and performed the linking. If there is a match then that counts towards false positive, which is equal to 75 individuals.*

*This gives us the following values for recall, FPR, and precision:*

*Recall = tp/(tp+fn) = 0.55*

*False positive rate = fp/(fp+tn) = 0.20*

*Precision = tp/(tp+fp) = 0.74*

*We have now added these calculations and associated text to the manuscript and supplementary information.*

*Excerpt From*

*Revised Manuscript*

*In main text:*

*We also calculated the precision as 74% and false-positive rate as 20% (see Supplementary Info).*

*In supplement:*

*Calculating precision, recall, and false positive rate*

*The attacker does not assume the victim is in the database. The assumption is that if the score of the top ranked match is different than the distribution of the scores for other individuals, then the top ranked match is the victim. If the score is not different from, say, the second ranked individual, the victim is not in the database. However, there might be significant matches that are incorrect.*

*Below how we calculated the metrics*

*1- We can correctly match the individual, who is in the database. This counts as a true positive, which is equal to 211 individuals.*

*2- The individual is in the database but we don’t have a match (i.e score that is different from the score of the second best match). This counts as false negative, which is equal to 171 individuals.*

*3- We removed the individuals from the database one by one and performed the linking. If there is no match then that counts towards true negative, which is equal to 307 individuals.*

*4- We removed the individuals from the database one by one and performed the linking. If there is a match then that counts towards false positive, which is equal to 75 individuals.*

*This gives us the following values for recall, FPR, and precision:*

*Recall = tp/(tp+fn) = 0.55*

*False positive rate = fp/(fp+tn) = 0.20*

*Precision = tp/(tp+fp) = 0.74*

It is not clear how authors calculate the significance levels. They refer to a previously defined gap measure but no references are given. Please also discuss here with notation.

*Response*

*We thank the reviewer for this comment. We did explain the calculation of gap in the methods section with reference to our previously published paper*. Below how we calculated gap and statistical significance with the notation from our previous publication*. We have now expanded the section we described gap and statistical significance by also avoiding duplication with our previous paper.*

*gap*

*To find the individual in database D that matches the query individual, we then rank all the L(i,query) scores for a given query individual in decreasing order as*

*where m is the total number of individuals in the attacked database. We denote the individual with the highest score as the queried individual. To assess the statistical robustness of this prediction, we defined a measure called gap, which is the ratio between the L(i,query) score of the first-ranked individual (max=L(i,query)1) and that of second-ranked individual (max2=L(i,query)2 and gap=max/max2). The goal is to determine how separated the matching individual is from the rest of the individuals in the cohort.*

*Statistical significance of gap*

*We estimated the empirical p-values for a particular gap value by linking a set of random genotypes that do not belong to a particular individual to the database, as follows.*

*We selected n random genotypes from a database of nearly 50 million genotypes (taken from 2,504 individuals in the 1000 Genomes database). n is the total number of genotypes that were linked to the database for the gap calculation.*

*We calculated the L(i,query) between these random n genotypes and every individual i in the database.*

*We calculated the gap as the ratio between the first-ranked and second-ranked L(i,query).*

*We repeated the above steps 1,000 times and obtained a distribution of random gap values.*

*The total number of random gap values that are equal to or greater than the real gap divided by 1,000 is the probability of observing the real gap value by chance.*

*We empirically calculated the probability of observing the real gap value or a larger gap value by chance by randomly subsetting n variants (the same number of variants as in predicted list for a given individual), performing the linkage attack, and calculating the associated gap value. If this p value is statistically significant, then the attacker can rely on the prediction.*

**Gürsoy, G. et al. Data sanitization to reduce private information leakage in functional genomics, Cell*

*Excerpt From*

*Revised Manuscript*

*In Online Methods:*

*Best match approach: For each individual j in the database, we find the intersection ScanSjD and calculate a linking score L(i,can)=t=0t=ScanSjD1log2 f(gt=1), where f(gt=1) is the ratio of individuals whose tth SNP has the heterozygous genotype (gt=1) to the total number of individuals in D [previously defined in 12]. To recover the genome for the known individual, we then rank all the L(i,can) scores for all genomes in D in decreasing order. We denote the genome with the highest score as the genome of the known individual with candidate SNPs. To assess the statistical robustness of this prediction, we used our previously defined gap measure, which is the ratio between the L(i,can) score of the first-ranked individual (max=L(i,can)1) and that of second-ranked individual (max2=L(i,can)2 and gap=max/max2). We further calculate the statistical significance of gap by generating random candidate SNPs (as many as the original candidate SNPs), perform the above attack one thousand times, and compare the real gap value against the distribution of random gap values.*

I do not understand why adding both ancestry and gender results in a lower performance increase. Please discuss the reason.

*Response*

*This is only because out of 382 individuals, only 20% (76 individuals) had non-European ancestry and 50% was the gender ratio.*

*Ancestry only helped for the linking of 20% of the individuals. The random chance of linking an individual to a database by knowing the gender increased the probability from 0.0004 to 0.0008.*

*We have now added sentences describing these reasons.*

*Excerpt From*

*Revised Manuscript*

*The reason behind these moderate increases are a- because the non-European individuals were only 20% of the 382 individuals, hence no additional information was gained for the majority of the individuals and b- the additional information gained by knowing biological sex is relatively low (-log2(0.5)) compared to the information obtained from a string of rare SNPs.*

The authors do not have a fully phased dataset, so the heterozygous SNP frequency calculation and filtering is an ad-hoc procedure. How sensitive is this attack to different filter thresholds?

*Author Response*

*We thank the reviewer for this comment. We have decomposed this question into two parts:*

*Heterozygous genotyping frequency: In order to calculate the heterozygous genotyping frequency, one does not have to have the phasing information. We can simply calculate heterozygous genotyping frequency of a SNP in a dataset by counting the number of individuals who have a heterozygous genotype for that SNP.*

*Filtering threshold: Below we detailed the logic behind this thresholding. We have also done testing by changing this threshold to show how this threshold affects the linking ability.*

*If a heterozygous SNP has a genotyping frequency smaller than 0.1, then it means that the alternative allele is present in a small number of individuals, which makes it very informative if we calculate the self information (>-log2(0.1)). Although this is very useful in linking individuals to a database when we know “for sure” that the alternative allele is present in the victim's genome. However, in our scenario, the attacker makes predictions of the presence of the rare allele purely based on the fact that if the gene has ASE or not. The attacker will more likely be wrong in guessing it than being right. If the attacker falsely predicts the presence of an alternative allele that is very rare in the population, the resulting linking score will be dominated by the contribution of this rare SNP and, in turn, will lead to linking the ASE to the wrong individual. Therefore these SNPs are removed from the list.*

*If a heterozygous SNP has a genotyping frequency larger than 0.5, then it means it is common in the population. Therefore, it’s contribution to the linking score is 1 (-log2(0.5)) or smaller. Adding this criteria to the predicted SNPs helps attackers weed out the non-informative SNPs from the list for a quicker calculation. Therefore these SNPs are removed from the list.*

*We have calculated how the thresholding affects the linking ability. We found that when there is no thresholding, the accuracy drops from 55% to 41%. When we only remove the rare ones (<0.1 genotyping frequency) and leave the ones with >0.5 genotyping accuracy, our accuracy is 55%, suggesting the removal of common SNPs do not affect the linking ability but makes the calculation faster by reducing the number of SNPs in the list. We have also tried other genotyping frequency thresholds to see the effect.*

*We have now explained this in the manuscript and added a table on how this criteria affects the overall linking ability.*

*Excerpt From*

*Revised Manuscript*

*In main text, online methods:*

*We then removed the SNPs that had 0.1< f(genotypeSNPi=1)< 0.5 from the overlap list (see Supplementary Information for the rationale).*

*In supplementary information:*

*Genotyping frequency threshold for SNP selection*

*If a heterozygous SNP has a genotyping frequency smaller than 0.1, then it means that the alternative allele is present in a small number of individuals, which makes it very informative if we calculate the self information (>-log2(0.1)). Although this is very useful in linking individuals to a database when we know “for sure” that the alternative allele is present in the victim's genome. However, in our scenario, the attacker makes predictions of the presence of the rare allele purely based on the fact that if the gene has ASE or not. The attacker will more likely be wrong in guessing it than being right. If the attacker falsely predicts the presence of an alternative allele that is very rare in the population, the resulting linking score will be dominated by the contribution of this rare SNP and, in turn, will lead to linking the ASE to the wrong individual. Therefore these SNPs are removed from the list.*

*If a heterozygous SNP has a genotyping frequency larger than 0.5, then it means it is common in the population. Therefore, it’s contribution to the linking score is 1 (-log2(0.5)) or smaller. Adding this criteria to the predicted SNPs helps attackers weed out the non-informative SNPs from the list for a quicker calculation. Therefore these SNPs are removed from the list.*

*We have calculated how the thresholding affects the linking ability. We found that when there is no thresholding, the accuracy drops from 55% to 41%. When we only remove the rare ones (<0.1 genotyping frequency) and leave the ones with >0.5 genotyping accuracy, our accuracy is 55%, suggesting the removal of common SNPs do not affect the linking ability but makes the calculation faster by reducing the number of SNPs in the list. Below are different threshold and the number of correctly individuals:*

*No threshold = 157/382 = 0.41*

*Remove only rare (<0.05) = 90/382 = 0.24*

*Remove only rare (<0.1) = 211/382 = 0.55*

*Remove only rare (<0.2) = 173/382 = 0.45*

*Remove only rare (<0.1) and common (>0.5) = 211/382 = 0.55*

What is the term "e" in the entropy? Also what is the purpose of using this method? Can't the authors already have a distribution from the linking scores?

*Author Response*

*“e” is the exponential.*

*Linking score is nothing but an approximation to the self-information of a set of SNPs in the database (linking score = -log(p(X)). By taking the exponential of the linking score, we are approximating the probability that this set of SNPs is matching to a set of SNPs in the database. This was adopted completely from the famous Netflix-IMDB linking attack paper by Narayanan and Schamtikov in 2012. We have cited this paper in our Online Methods, entropy approach section in our initial submission.*

*Since the linking score is calculated from the log of the probabilities, using the distribution of linking scores to calculate the probability wouldn’t be a correct estimation.*

*Excerpt From*

*Revised Manuscript*

*N/A*

**Second round of review**

**Reviewer 1**

The revision has adequately addressed my comments.

**Reviewer 2**

I thank the authors for the revisions made based on my comments. I have the following minor comments.

1. Please calculate the precision and recall values also for the HLA-removed scenario and when considering the top-20 matches.

2. Now reading the response, the formula for the entropy method should be c*e^(L(i, can)/\sigma) but written as c*e*(L(i, can)/\sigma). Please correct. Also Narayanan and Schamtikov paper is from 2008 but cited as 2012.

**Authors Response**

**Point-by-point responses to the reviewers’ comments:**

*We have also added new calculations per reviewer 2's comments. Specifically, we have added precision and FPR values for the cases when we remove the HLA genes and when we remove top 20 genes. The numbers can be found in the main manuscript and it refers to the additional file for the details. We have also corrected the entropy formula.*
